# Prevalence and environmental determinants of cutaneous leishmaniasis in rural communities in Tigray, northern Ethiopia

**DOI:** 10.1371/journal.pntd.0007722

**Published:** 2019-09-26

**Authors:** Mekonnen Yohannes, Zerihun Abebe, Eline Boelee

**Affiliations:** 1 Department of Medical Parasitology & Entomology, School of Medicine, College of Health Sciences, Mekelle University, Mekelle, Ethiopia; 2 Dermatovenerology unit, School of Medicine, College of Health Sciences, Mekelle University, Mekelle, Ethiopia; 3 Deltares, Utrecht, the Netherlands; Centro de Pesquisa Gonçalo Moniz-FIOCRUZ/BA, BRAZIL

## Abstract

**Background:**

In Ethiopia guidelines for diagnoses and treatment of leishmaniases are available, but only a few hundred people are diagnosed and receive treatment. A field study has been carried out to determine the status and environmental determinants of cutaneous leishmaniasis (CL) and assess the degree of awareness of the rural communities in affected areas in Tigray, northern Ethiopia.

**Methodology / Principal findings:**

Following a reconnaissance survey that identified endemic foci, a cross sectional door-to-door survey was conducted in 2009 in five rural communities around the towns of Adigrat and Hagereselam in Tigray. In total 9,622 residents of 1,721 households were clinically screened and household heads interviewed regarding the determinants of infection. The χ^2^ test and logistic regression were used to determine differences in prevalence between localities, age and sex, and to identify environmental determinants of infection.

The overall prevalence of localized CL was 2.3% (highest 4.7%), with marked inter-village differences. Another 20.9% had scars from previous infections. While risk was sex-independent, prevalence was significantly higher in the 0–9 (4.5%) and 10–19 (2.5%) age groups and predominantly involved the face (82.1%) and upper limbs (13.1%). Nearly 11% of the households had one or more cases of CL and this was associated with proximity to hyrax habitats. All interviewees were knowledgeable about the lesions but ignorant of the disease’s mode of transmission and its association with hyraxes.

**Conclusions:**

The study established that CL is an important public health problem in the study communities, and has been so for a while, as demonstrated by the widespread presence of scars. CL in Tigray appeared to be predominantly of zoonotic nature, mainly transmitted in peri-domestic habitats in proximity to hyrax habitats. Integrated interventions, including awareness creation, are highly recommended.

## Author summary

Cutaneous leishmaniasis (CL) is a skin infection, transmitted by sandflies. It is most common in Ethiopia, but so far only a few hundred people have received treatment. Five rural villages in Tigray Region, in the north of Ethiopia, were visited to assess the status and determinants of CL. In a door-to-door survey 9,622 residents of 1,721 households were examined and interviewed. A total of 222 had active lesions, an average prevalence of 2.3% CL. Children (up to 9 years old) and teenagers (age 10–19) were more affected than other groups. Most active lesions were found in the face and on arms. Almost 11% of the households had one or more cases of CL and this was associated with proximity to habitats of hyrax, intermediate hosts of the disease. A total of 2009 people (20.9%) showed scars from earlier infections. The findings show how widespread the disease is in the north of Ethiopia and provide some first insights into the environmental factors that influence transmission.

## Introduction

Ethiopia is among the 98 leishmaniasis endemic countries of the world with both the visceral and cutaneous forms prevalent in the country [1]. An estimated 0.2 to 0.4 million visceral leishmaniasis (VL) cases and 0.7 to 1.2 million cutaneous leishmaniasis (CL) cases occur each year globally [2]. CL due to *Leishmania aethiopica* has long been the most widespread skin disease in the highlands of Ethiopia [3,4,5] principally affecting the poor rural communities. A recent study indicated that an estimated 29 million people are at high risk of CL in the central highlands of the country [6]. Estimates of the annual incidence range from 20,000 to 50,000 cases yearly [7,8], but only a few hundred cases are actually reported [8]. Stable foci are maintained by hyraxes (small herbivorous mammals *Procavia capensis* and *Heterohyrax brucei*), and the parasite is transmitted among hyraxes and humans by female phlebotomine sandflies (*Phlebotomus longipes* and *P*. *pedifer*) that feed mainly on hyraxes and share their habitat [9,10,11]. There are three clinical forms of CL due to *L*. *aethiopica*: localized CL (LCL), mucosal cutaneous leishmaniasis (MCL), and diffuse cutaneous leishmaniasis (DCL). Although not fatal, persistent LCL, MCL and DCL are disfiguring [12] and may bring long-term psycho-social problems, particularly in young women.

CL is the most neglected even among the neglected tropical diseases (NTDs) in the country. Exact figures on the magnitude of CL in Ethiopia are lacking both nationally and by regional state. The first guideline for diagnosis, treatment and prevention of VL was produced in 2006, and updated with the inclusion of CL in June 2013. However, only very few health centres (eight) diagnose leishmaniasis and as of 2014, only 342 CL cases were treated in VL treatment centres [13]. Diagnosis of CL involves clinical assessment and confirmation with microscopic examination of skin lesion sample. Antimonials are approved for CL treatment in the selected health centres, but most cases are treated traditionally using plants and local application of heat (with hot iron or charcoal fire as per local practice) [8]. There is no leishmaniasis vector control program. Distribution of insecticide-treated nets (ITNs) and insecticide spraying in the context of malaria control may have some impact on phlebotomines in lowland localities where VL is also endemic. On the whole, there is limited evidence, nor control efforts of CL in the country. Outbreaks of CL are not uncommon [14].

The risk of HIV / CL co-infection is also a serious threat as it increases the burden of CL by causing severe forms that are more difficult to manage [15]. Absence or limited access to diagnosis and treatment for CL further increases the urgency for epidemiological surveillance of the disease in the country.

Reports pertaining to CL in Ethiopia date back to 1913, but the disease appears to be around much longer considering the presence of vernacular names in every language where the disease is endemic. Despite its long recognized endemicity [1,3], information on the epidemiology of CL in Ethiopia is still incomplete [16]. Tigray is one of the regions in northern Ethiopia where the status of CL is still unknown. This is mainly due to absence of epidemiological field studies in the region. Due to absence of diagnosis and drugs to treat CL, even reports from health facilities indicating its mere presence in the region have been scarce [15]. This study was initiated following our observation of a steady flow of severe CL cases to a newly established (2005) Italian dermatological unit that started to provide treatment at Ayder Referral and University Teaching Hospital in Mekelle, the capital of the region. With the aim to identify active CL foci and map the pattern of distribution of the disease in the region, a large-scale study has been in progress since late 2008. Part of this study was a door-to-door survey (see Supporting information S1 STROBE checklist) undertaken in five rural communities of Tigray to assess the extent of CL presence and in high risk areas identify potential environmental risk factors, the results of which are presented here.

## Methods

### Ethics statement

The study was approved by the Research Ethics Review Committee (RERC) of College of Health Sciences at Mekelle University under reference number CHS/790/DN-16. Permission from the district and respective village authorities was also obtained. Informed oral consent was obtained from the head of the household selected for the study and for those with active lesions signed consent was sought from the guardians. The data were anonymised before analysis by replacing birth dates by age range and household details by name of the subdistrict.

### Study area

**Fig 1:**
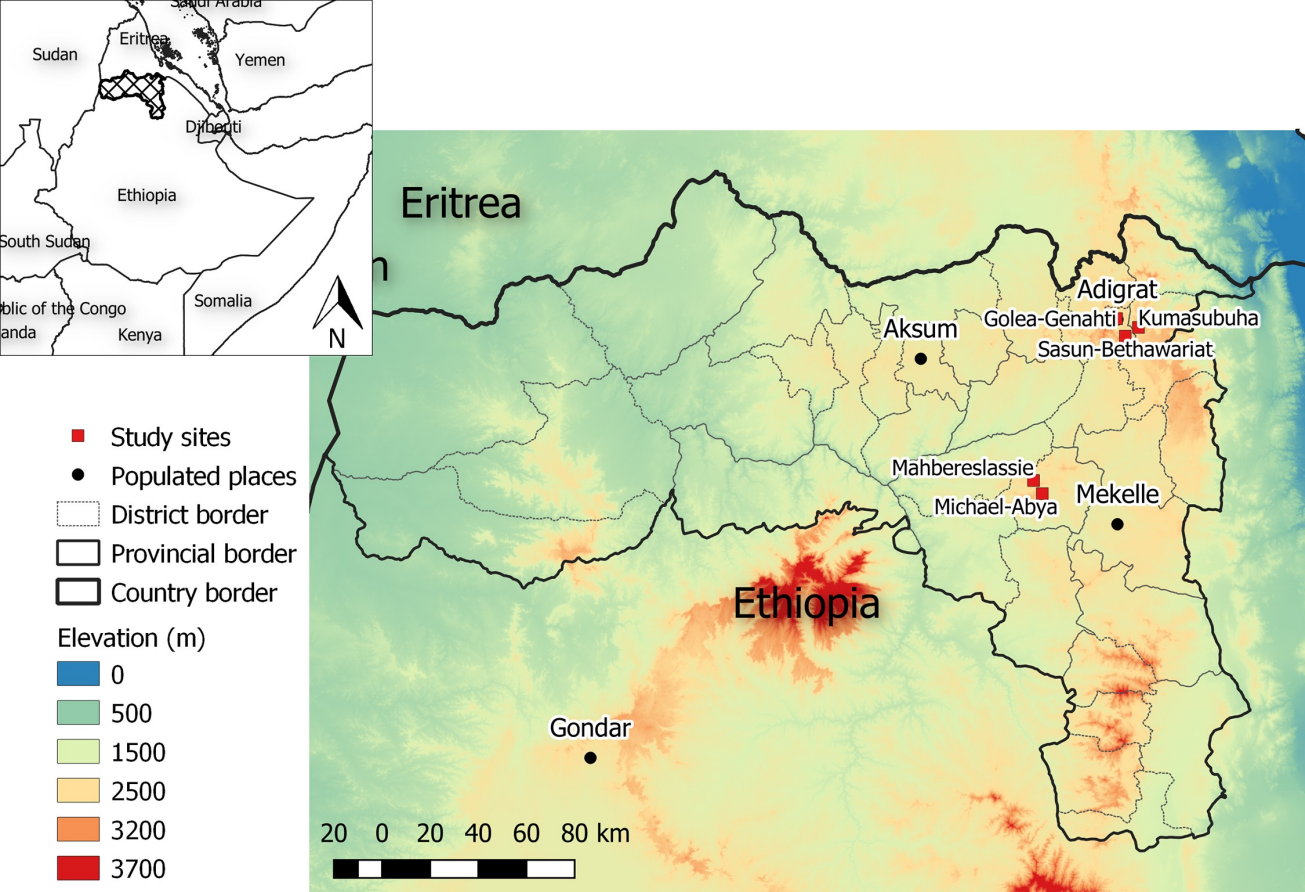
Location of study sites. Map prepared with QGIS version 3.4 (https://qgis.org/en/site/), based on open source maps with elevation [19] and administrative borders [20,21].

The study was carried out in March and April of 2009 in villages around the towns of Adigrat and Hagereselam in the Tigray National Regional State in northern Ethiopia. The region covers a surface area of about 50,000 km^2^ and borders with the Sudan in the west and Eritrea in the north (Fig 1). Its nearly 4.3 million people are predominantly rural (80.5%) and engaged in subsistence rain-fed agriculture [17]. The region has a diverse topography, with peak highlands (8%), midlands (39%) and lowlands (53%). Its altitude varies from about 200 meters above sea level (masl) in the north east to almost 4165 masl in the south west. The climate is semi-arid, and the rainfall pattern is mainly unimodal (June to September) but erratic (200 to >1000 mm annually). The regional average annual temperature is about 18.0°C but varies greatly with altitude [18].

The first study site lies to the east of the town of Adigrat. Adigrat is the administrative centre of the eastern zone, located at about 900 km north of Addis Ababa and 120 km north of Mekelle, the regional capital. Locally known for its CL endemicity, the study area comprises of three adjacent subdistricts (kebeles, peasant associations) demarcated by huge gorges: Golea-Genahti, Sasun-Bethawariat, and Kumasubuha (Table 1). The altitude of the study sites ranges from 2248–2650 masl with mild to high temperatures and rainfall is on average 400 mm per year. The area is characterized by deeply incised plateaus, dominated by limestone and sedimentary rock formations providing ideal habitats for hyraxes. The area has moderate to high density population with heavily deforested plains, predominantly scattered bush, and acacia trees. Cactus grows wild in the backyards of most homes. The soils are sandy and of low fertility.

**Table 1:** Description of the study sites (subdistricts within their respective districts).

Study site	Coordinates	Altitude (masl)*	No HHs*	Climate			
				Annual average rainfall (mm)	Temperature (°C)		
					Tmin*	Tmax*	Average
**Ganta-Afeshum district**							
Golea-Genahti	14°17'N & 39°28'E	2248–2579	1246	400 (<400–600)	7–9	23–26	15–18
Sasun-Bethawariat	14°13'N & 39°30'E		800	400 (<400–600)	7–9	23–26	15–18
**Saesie-Tsaedaemba district**							
Kumasubuha	14°15'N & 39°33'E	2250–2650	1067	400 (<400–600)	7–9	23–26	15–18
**Degua-Tembien district**							
Mahbereslassie	13°40'N & 39°09'E	2256–2700	425	700 (600–800)	7–9	23–26	15–16
Michael-Abya	13°37'N & 39°11'E	2340–2574	400	700 (600–800)	7–9	23–26	15–16

* masl = meters above sea level; No HHs = Number of households; Tmin = minimum temperature; Tmax = maximum temperature.

The second study site is located around the market town of Hagereselam (altitude 2650 masl), the administrative centre of Degua-Tembien district 50 km west of Mekelle. It consists of villages belonging to two subdistricts: Mahbereslassie and Michael-Abya (Table 1). The district is about 1033 km^2^ with an average population density of about 108 persons/km^2^ [17]. The area has a stepped landscape where flats alternate with steep escarpments. As part of an environmental rehabilitation and reforestation programme nearly 10% of the total area of the district is closed off from people and livestock. Such ‘exclosures’ are mostly located on steep and degraded slopes. The climate of the district is locally classified as Dega (lower highland; 2200–2800 masl), receiving an average annual rainfall of around 700mm. The mean annual temperature is about 15–16°C.

The epidemiological status of leishmaniasis in the study area is not known, owing to the absence of field-based research and limited access to treatment. Based on university hospital records between February 2005 and February 2009, 21 cases of CL were reported from the Adigrat area and 23 cases from the study villages around the town of Hagereselam. VL has never been reported from these highland localities.

### Study design and sampling

The subdistrict villages were selected based on information from medical records of Ayder Referral Hospital and personal experience. In absence of reliable boundary and demographic data at the time, the largest possible sample size was sought by considering every other household in each selected subdistrict. Accordingly, a total of 1721 households were surveyed from the study areas, namely: 437 in Golea-Genahti, 378 in Sasun-Bethawariat, 511 in Kumasubuha, 210 in Mahbereslassie, and 185 in Michael-Abya.

Household members’ socio-demographic, clinical and household data as well as information pertaining to environmental determinants, knowledge on mode of transmission and prevention methods of CL were recorded by a regularly supervised research team consisting of trained health officers guided by a community member knowledgeable on CL. The questionnaire is added as supporting information (S1 Text).

The skin lesions of CL are well identified by the community in its vernacular name “Gizwa”. The required information was sought from the household head or eligible adult present during the visit. Information, collected at each household, included housing type and demographic variables such as age, sex, occupation, duration of residence, and number of animals owned (livestock, dogs). We also observed and recorded the geographic features of the household, including proximity to caves, gorge, and hyrax habitats.

Household heads were also asked for the presence of members with active skin lesions or scars due to CL or other causes. When present, members with evidence of CL were summoned and examined for lesions and scars. For each case, information was recorded on the number, type (LCL, MCL, and DCL) and location of lesions and scars, as well as determinants of infection, such as history of travel and outdoor sleeping. Healed lesions that are depressed, none-or hypo-pigmented, but shiny at the periphery and with rubbery borders, were diagnosed as past CL and referred to as ‘scars’. Papular, nodular or ulcerative lesions with or without satellite lesions and mostly located in uncovered parts of the body were clinically diagnosed as LCL (‘lesions’). Lesions involving the mucosa was considered as MCL while multiple non-ulcerative nodular lesions, often bigger in size from those lesions of LCL were operationally defined as DCL. People with lesions were considered as cases of active CL and this was used to calculate prevalence. Samples of skin snips (n = 51) were taken from a subsample of active cases, smeared on microscope slides, stained with Giemsa and examined for presence of *Leishmania* amastigotes. The remaining active cases were referred for treatment to Ayder Referral Hospital in the capital and reports of tissue smears of those who visited the hospital were sought from the laboratory records. This confirmed that active CL was caused by *Leishmania* parasites, most probably *L*. *aethiopica*.

### Data analysis

Frequencies and proportions were used for the descriptive analysis of the data. The χ^2^ test was used to determine any statistically significant difference in disease prevalence between age groups, sexes and areas and a P-value <0.05 was considered significant. Any association between the presence of lesions and environmental and host factors was sought using logistic regression. As the flight range of most sandflies is estimated at 300 m from their breeding sites [22], this distance was taken as the cut off point for the analysis of environmental factors such as proximity to caves, gorges and hyrax habitats. The data were analysed using SPSS (Statistical Package for Social Sciences, version 16). The full data set is added as supporting information (S1 Data).

## Results

### Prevalence of CL

A total of 9622 inhabitants in 1721households were surveyed in the selected subdistricts. Males (50.01%) and females (49.99%) were represented almost equally. All study participants resided in the area for more than three years and none of them travelled out of their area within the last 6 months during the study period. However, three people with active CL claimed to have travelled to neighbouring CL endemic subdistricts prior to the appearance of lesions. Prevalence of active CL was 2.3%, with an additional 20.9% of the population showing scars. All active lesions observed during the study were of the localized type (LCL). Of the 1721 households sampled, nearly 11% (188) had one or more cases of active CL, with a total of 60% that had either lesions or scars. Of the 188 households with active CL, 159 (84.6%) households had one case, 25 (13.3%) had two cases, and 4 (2.1%) had three or more cases.

There was a marked difference in prevalence between the study localities. The highest prevalence (active lesions) was observed in Mahbereslassie subdistrict (4.7%) in Degua-Tembien district and in Kumasubuha (2.7%) in Saesie-Tsaedaemba district (Table 2). Most scars, indicative of past infections, were found in Kumasubuha (34.4%). Statistically significant differences were observed between the five study sites, both in the prevalence of lesions (χ2 = 45.860; df = 4; p < 0.001) and scars (χ2 = 628.080; df = 4; p < 0.001).

**Table 2:** Distribution of CL among subdistricts, sex and age groups in Tigray, northern Ethiopia (2009).

Categories			Lesion		Scar		Total	
		N*	n*	%	n	%	n	%
Subdistrict	Golea-Genahti	2429	46	1.9	351	14.5	397	16.3
	Sasun-Bethawariat	2049	32	1.6	452	22.1	484	23.6
	Kumasubuha	3017	81	2.7	1037	**34.4**	1118	37.1
	Mahbereslassie	1154	54	**4.7**	131	11.4	185	16.0
	Michael- Abya	973	9	0.9	38	3.9	47	4.8
Sex	Male	4812	119	2.5	1003	20.8	1122	23.3
	Female	4810	103	2.1	1006	20.9	1109	23.1
Age	0–9	2367	107	**4.5**	275	11.6	382	16.1
	10–19	3016	75	2.5	846	**28.1**	921	30.5
	20–29	1222	10	0.8	341	27.9	351	28.7
	30+	3017	30	1.0	547	18.1	577	19.1
	**Overall**	**9622**	**222**	**2.3**	**2009**	**20.9**	**2231**	**23.2**

* N = number examined from each category; n = number with active or past lesions.

In the present study, males showed slightly higher rates of active lesions (2.5%; 119/4812) than females (2.1%), but the difference was not statistically significant (χ^2^ = 1.17, df = 1, p = 0.762). Age specific active prevalence was significantly higher in the 0–9 years olds (4.5%; χ2 = 86.96; df = 3, p < 0.001) than for those more than 10 years old (average 1.6%; χ2 = 86.96; df = 3, p < 0.001). Of those with active lesions, 71.6% (159/222) were under 15 years of age and nearly 20% (44/222) were less than 6 years. The youngest person with active CL was eleven months old.

**Fig 2:**
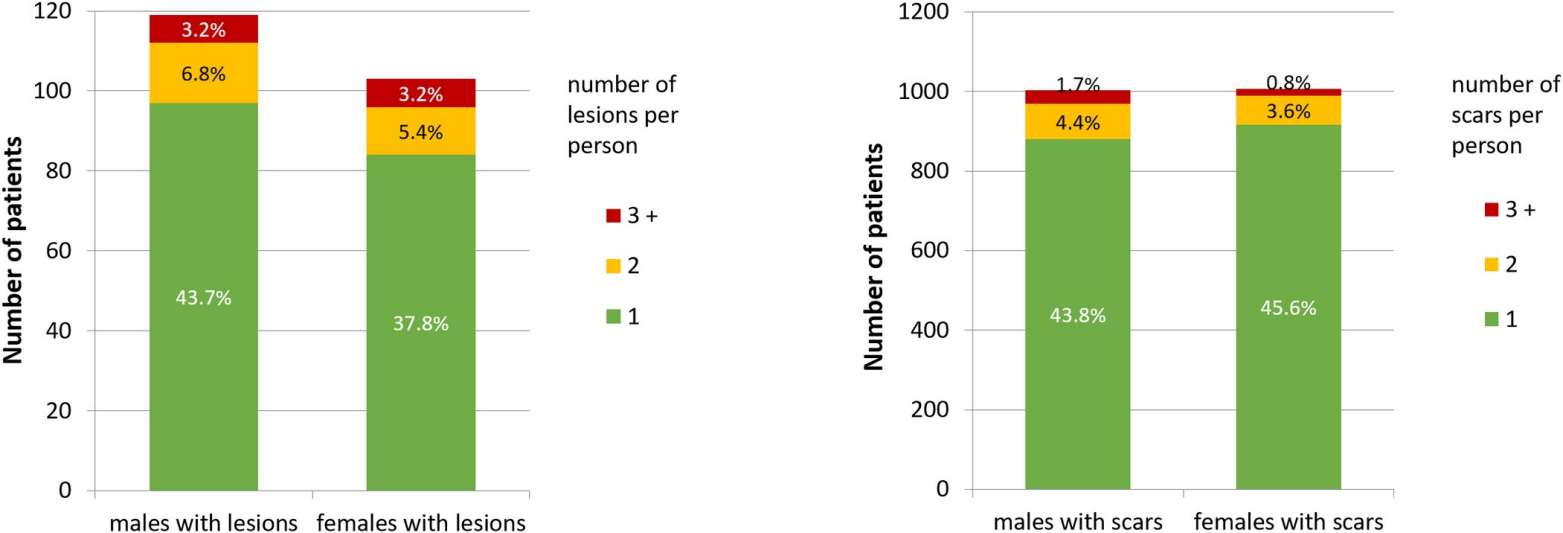
Number and percentages of male and female patients with active lesions and scars in five subdistricts around the towns of Adigrat and Hagereselam in Tigray, northern Ethiopia (2009).

The majority (82.1%; 207/252) of lesions were found on the face, in which the cheeks and nose were the most affected (Table 3). A similar pattern of distribution was observed with regard to scars. The number of lesions or scars per individual ranged from 1 to 7 (Fig 2). Nearly 90% (199/222) of active CL cases had single lesions and 93.5% (1879/2009) of those with healed lesions had single scars. Most of the single lesions (90%) were of the ulcerative type, with indurated margins and necrotic base, appearing as reddish plaques with irregular borders covered by a firmly adherent crust. Nearly 82% of the active lesions had developed less than two years ago. Out of the 51 active CL skin scraping smears examined, amastigotes were found in 31 (60.8%) of them. All tissue smears, of twenty individuals with lesions who visited the hospital, were found to be positive for amastigotes raising the total percentage to 71.8%.

**Table 3:** Distribution over body parts* of active lesions and scars of CL cases from five subdistricts around the towns of Adigrat and Hagereselam in Tigray, northern Ethiopia (2009).

Location	Lesion		Scar		Total	
	n	%	n	%	n	%
Cheek	127	**50.6**	908	**42.1**	1035	43.0
Nose	41	16.3	418	19.4	459	19.1
Forehead	27	10.8	202	9.4	229	9.5
Ear	10	4.0	123	5.7	132	5.5
Lip	1	0.4	2	0.1	3	0.1
Chin	1	0.4	0	0	1	0.0
**Total face**	**207**	**82.1%**	**1653**	**76.7%**	**1859**	**77.3%**
Arm	33	13.1	403	18.7	436	18.1
Leg	9	3.6	94	4.4	103	4.3
Other	3	1.2	5	0.2	8	0.3
**Total**	**252**	**100**	**2155**	**100**	**2405**	**100**

*Multiple lesions on a single individual on different location of body parts were counted with their respective locations. n = number of individuals with active lesions / scars on the indicated body parts of the participants.

### Environmental and host factors

Several environmental and host related factors were assessed for their association with active CL cases using univariate and multivariate logistic regression (Table 4). Significant variations were observed in prevalence of active CL among age groups, study villages, and physical features of household location, including the presence of hyraxes, caves, and gorges within 300 m of the residence. Individuals in the age group of 0–9 years were nearly five times (OR = 4.71; 95% CI: 3.13–7.1) and those aged 10–19 years were 2.5 times (OR = 2.54; 95% CI: 1.66–3.89) more likely to have CL compared to individuals in the age group of 30 years and above. However, those in the age group 20–29 years were 18% (OR = 0.82; 95% CI: 0.4–1.69) less likely to have active CL. Livestock ownership (OR = 1.65; 95% CI: 1.001–2.71), presence of hyraxes (OR = 4.15; 95% CI: 2.64–6.53), gorges (OR = 3.63; 95% CI: 2.39–5.52), and caves (OR = 3.22; 95% CI: 2.32–7.47) in the vicinity were highly associated with the presence of active CL. Accordingly, participants who lived near hyrax colonies were 4.2 times and those living near caves were 3.2 times more likely to be infected by CL compared to those living far away. Similarly, those living within 300 m of a gorge were found to be 3.6 times more likely to get CL than those far away. Besides, those who slept outdoor were two times (OR = 2.04; 95% CI: 1.29–3.21) more likely to get CL than those who slept inside. Similarly, individuals residing in households with livestock were found to be 65% times more likely to be infected with CL than those without.

**Table 4:** Summary of univariate and multivariate logistic regression analysis pertaining to environmental and host factors associated with active CL prevalence* in study sites around the towns of Adigrat and Hagereselam in Tigray, northern Ethiopia (2009).

Environmental Factors	N*	CL +*	Univariate			Multivariate		
		n (%)	OR*	CI* (95%)	P-value	AOR*	CI* (95%)	P-value
Gorge near home (300m)								
NO	2992	25 (0.8)	1			1		
YES	6630	197 (3.0)	3.63	2.39–5.52	< 0.001	2.13	1.37–3.33	0.001
Cave near home (300m)								
NO	4344	46 (1.1)	1			1		
YES	5278	176 (3.3)	3.22	2.32–4.47	< 0.001	1.60	1.07–2.38	0.022
Hyrax near home (300m)								
NO	2866	21 (0.7)	1			1		
YES	6756	201 (3.0)	4.15	2.64–6.53	< 0.001	2.47	1.49–4.12	0.001
**Host factors**								
Sleeping outdoor								
NO	8999	194 (2.2)	1			1		
YES	623	28 (4.5)	2.14	1.43–3.20	<0.001	1.73	1.14–2.63	0.010
Cattle presence								
NO	1147	17 (1.5)	1			1		
YES	8475	205 (2.4)	1.65	1.01–2.71	0.047	0.97	0.58–1.62	0.895
Age group								
0–9	2367	107 (4.5)	**4.71**	3.13–7.09	<0.001	4.58	3.03–6.90	0.000
10–19	3016	75 (2.5)	2.54	1.66–3.89	<0.001	2.53	1.65–3.88	0.000
20–29	1222	10 (0.8)	0.82	0.40–1.69	<0.001	0.81	0.39–1.67	0.570
30+	3017	30 (1.0)	1			1		
**Subdistricts**								
Michael-Abya	973	9 (0.92)	1			1		
Golea-Genahti	2429	46 (1.9)	2.1	1.01–4.24	0.047	1.66	0.76–3.61	0.205
Sasun-Bethawariat	2049	32 (1.6)	1.7	0.81–3.57	0.162	1.25	0.56–2.77	0.586
Kumasubuha	3017	81 (2.7)	2.96	1.48–5.91	0.002	1.84	0.86–3.94	0.118
Mahbereslassie	1154	54 (4.7)	**5.36**	2.58–10.7	<0.001	2.82	1.31–6.08	0.008

* N = number of subjects from each category; n = number with active lesions; CL+ = those with active lesions; OR = crude odds ratio; AOR = Adjusted odds ratio; CI = Confidence interval.

When compared to those residing in Michael-Abya subdistrict, inhabitants living in Mahbereslassie were found to be nearly 5.3% (OR = 5.26; 95% CI: 2.58–10.71) times more likely to be infected with CL; in Kumasubuha this was 3% (OR = 2.96; 95% CI: 1.48–5.91), in Golea-Genahti 2.1% (OR = 2.1; 95% CI: 1.01–4.24), and 70% (OR = 1.70; 95% CI: 0.81–3.57) in Sasun-Bethawariat subdistrict (Table 4).

Except for livestock ownership, multivariate logistic regression analysis also showed the significant effect of the environmental and host factors on the odds of being positive for CL (Table 4). Accordingly, the age groups 0–9 years (AOR = 4.67; 95% CI: 3.10–7.04; p< 0.001) and 10–15 years (AOR = 2.48; 95% CI: 1.62–3.81; p<0.001), outdoor sleeping (AOR = 2.67; 95% CI: 1.17–2.67; p = 0.007) and location of residences in proximity to caves (AOR = 2.62; 95% CI: 1.26–2.62; p = 0.001), gorges (AOR = 2.43; 95% CI: 1.57–3.75; p < 0.001) and hyrax habitats (AOR = 2.35; 95% CI: 1.43–3.86; p = 0.001) was highly associated with the presence of active CL.

### CL awareness of respondents

All interviewed household heads were aware of CL and the great majority (98.7%) identified CL lesions. However, they were invariably ignorant of the disease’s association with hyraxes and its mode of transmission. Nearly all (99.8%) respondents claimed that CL is treated using traditional medicine, such as herbs, holy water (“Tsebel”) and local heat application, in that order (Table 5). Only 4 (0.2%) of the participants claimed the presence of modern treatment for CL. In some villages, farmers made use of the huge piles of hyrax pellets as manure, yet, all the interviewed said that hyraxes eat crops and vegetables and so hyraxes are considered an agricultural pest.

**Table 5:** Knowledge on CL among study participants (household heads) from five subdistricts around the towns of Adigrat and Hagereselam in Tigray, northern Ethiopia (2009).

Question		Frequency	%
Ever heard of CL?	Yes	1721	100
	No	0	0
CL present in your village?	Yes	1721	100
	No	0	0
Can you identify CL from other skin diseases?	Yes	1699	98.7
	No	22	1.3
Which body part is most affected?	Face	1721	100
	Arms	0	0
	Legs	0	0
	Other	0	0
Mode of transmission?	Yes	0	0
	No	1721	100
Relation of CL with hyraxes?	Yes	0	0
	No	1721	100
Source of CL treatment?	Traditional treatment	1717	99.8
	Modern treatment	4	0.2
Type of traditional treatment used	Herbs	1189	69.1
	Hot metal / coal	40	2.3
	Holy water / mud (Tsebel)	488	28.4

## Discussion

The present study revealed that the localities around the towns of Adigrat and Hagereselam are important CL foci in the region. All cases were of the localized type (LCL) and occurred mostly in the face, with the cheeks in particular. *Leishmania aethiopica* should be the etiologic agent as in a follow-up study conducted in a neighbouring subdistrict it was isolated as the main *Leishmania* species causing CL in the area [23], as in other parts of the country. The overall prevalence rate of active LCL in the study communities ranged from 0.9–4.7%, for scars this was 3.9–34.4% (Table 2). This is indicative of the public health significance of CL in these areas previously and currently. Despite good awareness and recognition of CL, understanding the mode of transmission lacked. This may have contributed to the high prevalence of the disease in the study villages and holds a risk of further spread in the future, since no active detection and treatment strategies are in place. Moreover, knowing that CL is transmitted by biting sandflies does not necessarily lead to adequate prevention strategies [24]. Integration with malaria control by distribution of ITNs and indoor residual spraying is unlikely as CL is predominantly prevalent in the malaria free highland areas.

The observed absence of gender sensitivity to infection by CL is consistent with several studies conducted elsewhere in the country. The predominance of CL in the young (0–9 years old) is also well known and resonates with countrywide data (e.g. [25,26], who found 8.5% and 7.1% respectively). In established endemic areas, CL prevalence typically increases with age up to 15 years, after which prevalence levels off, presumably because of the acquisition of immunity. In this study, the occurrence of the disease almost in equal proportion in both sexes, with large numbers of women and children infected, including those under one year, probably reflects that CL transmission may have occurred in peri-domestic habitats, where sandfly exposure is most equally distributed among individuals. This also explains why sleeping outdoors is an important (peri-domestic) risk factor. This is further corroborated by the fact that *Phlebotomus longipes*, the proven vector of CL in the Ethiopian highlands [9], was collected both from indoor and predominantly outdoor locations (compounds of houses) in a follow-up study conducted in the neighbouring subdistrict [23]. In peri-domestic settings, sandflies rest in cool, dark and humid corners of animal shelters or human dwellings [27,28]. As with rodent burrows, peri-domestic areas also provide ready access to bloodmeals in addition to shelter and suitable breeding grounds in decaying organic matter (manure). In line with previous reports from different parts of the country [9,23,29,30,31], the presence of CL cases in households was closely associated with the presence of hyrax colonies in the vicinity, indicating that the disease is mainly of zoonotic nature. The intimate ecological association of rocky hyraxes and the sandfly species *Phlebotomus longipes* and *P*. *pedifer*, the proven vectors of *L*. *aethiopica* induced CL in Ethiopia, is well established [30,31]. Two species of hyrax (*Procavia capensis* and *Heterohyrax brucei*) are the widely incriminated reservoir hosts of *L*. *aethiopica* in the country [30]. Livestock was also a risk factor, as *P*. *longipes* readily feeds on cows [9] and manure provides breeding habitat.

In the present study, some of the most important environmental determinants for CL occurrence were location of households near gorges and on rocky hillsides and presence of caves nearby. This is consistent with previous reports by Ashford [9] and Lemma et al. [3], who pointed out that gorges and escarpments, rock cliffs and mountainous areas constitute favourable environments for the reservoir host (hyrax). These features result in steep slopes, identified as a risk factor for CL by Seid et al. [6]. The presence of livestock and their manure further creates favourable breeding grounds for the sandfly vectors in peri-domestic environments.

Overall, this study establishes that important foci of CL exist in Tigray, northern Ethiopia, with children and young adults being the most affected. The data further highlight that the disease is predominantly of zoonotic nature, and mainly transmitted in peri-domestic habitats where hyraxes prevail in the vicinity. Apart from their role as reservoirs of CL, the status of hyraxes as agricultural pests needs to be determined. This offers perspectives for environmental transmission control complementing minimal efforts in passive and active case detection, treatment, reporting, and data analysis. Reservoir control, i.e. small-scale eradication of hyraxes in the proximity of dwellings, would be effective, especially combined with fogging of their habitats to reduce sandfly densities. Vector control alone is unlikely to be effective. In addition, regular education to children and adults on the transmission and prevention of CL is recommended. All control efforts should be evaluated periodically, with their impact on incidence, building on essential active case detection and monitoring.

Finally, although the study was carried out in 2009, we believe that our results still pertain to the present situation of CL in Ethiopia as there has been no progress in advancing treatment or prevention activities. Following our study, an awareness creating international consultative meeting was held by WHO and Federal Ministry of Health in 2011 and the first National Neglected Tropical Disease (NTD) Master Plan was launched In June 2013, to achieve WHO NTD elimination and control targets by 2020. As of September 2015, although the Federal Ministry of Health has managed to mobilize support to implement mass drug administration in 84% - 100% of the endemic districts for other NTDs (trachoma, onchocerciasis, lymphatic filariasis, soil-transmitted helminths and schistosomiasis), there has been no progress in advancing treatment or prevention activities in CL, owing to the absence significant domestic or international donors to support CL intervention activities. A follow-up study in 2013 (not part of this investigation) and further studies in progress indicate an even greater magnitude both in terms of spread and in level of prevalence of CL in the region [23]. Unfortunately, today CL remains neglected even among the NTDs.

## Supporting information

S1 Checklist STROBE checklist cross-sectional studies. This is the STROBE Statement, a checklist of items that should be included in reports of cross-sectional studies.
(DOC)

S1 Text Household questionnaire for mapping cutaneous leishmaniasis in Tigray. The form contains an introduction with basic details, a section for information on the household members, and a section with specific questions.
(DOC)

S1 Data Anonymised characteristics of 9622 inhabitants in 1721 surveyed households in Tigray, northern Ethiopia (2009). The data used in the analyses include subdistrict, sex, age group, history of CL, presence and location of lesions and environmental characteristics.
(XLSX)
