# Supplementary material for: Prevalence and environmental determinants of cutaneous leishmaniasis in rural communities in Tigray, northern Ethiopia
Source: PLoS Negl Trop Dis. 2019 Sep 26;13(9):e0007722. doi: 10.1371/journal.pntd.0007722 (PMC6782111; doi:10.1371/journal.pntd.0007722)
Supplement: S1 Checklist — This is the STROBE Statement, a checklist of items that should be included in reports of cross-sectional studies. (DOC) [file pntd.0007722.s001.doc]

**S1 Checklist: STROBE checklist cross-sectional studies**

Prevalence and environmental determinants of cutaneous leishmaniasis in rural communities in Tigray, northern Ethiopia

|  | Item No | Recommendation | In manuscript |
| --- | --- | --- | --- |
| **Title and abstract** | 1 | (*a*) Indicate the study’s design with a commonly used term in the title or the abstract | Title page, title. |
|  |  | (*b*) Provide in the abstract an informative and balanced summary of what was done and what was found | Title page, abstract. |
| Introduction | | |  |
| Background/ rationale | 2 | Explain the scientific background and rationale for the investigation being reported | Introduction, paragraph 1, 2, 3 (first part) |
| Objectives | 3 | State specific objectives, including any prespecified hypotheses | Introduction, paragraph 3 (last part) |
| Methods | | |  |
| Study design | 4 | Present key elements of study design early in the paper | Introduction, paragraph 3 (last sentence)  Methods: Study design and sampling |
| Setting | 5 | Describe the setting, locations, and relevant dates, including periods of recruitment, exposure, follow-up, and data collection | Methods: Study area |
| Participants | 6 | (*a*) Give the eligibility criteria, and the sources and methods of selection of participants | Methods: Study design and sampling, paragraph 1 |
| Variables | 7 | Clearly define all outcomes, exposures, predictors, potential confounders, and effect modifiers. Give diagnostic criteria, if applicable | Methods: Study design and sampling, paragraph 2, 3 |
| Data sources/ measurement | 8* | For each variable of interest, give sources of data and details of methods of assessment (measurement). Describe comparability of assessment methods if there is more than one group | Methods: Study design and sampling, paragraph 2, 3 |
| Bias | 9 | Describe any efforts to address potential sources of bias | Methods: Study design and sampling, paragraph 1, 2 |
| Study size | 10 | Explain how the study size was arrived at | Methods: Study design and sampling, paragraph 1 |
| Quantitative variables | 11 | Explain how quantitative variables were handled in the analyses. If applicable, describe which groupings were chosen and why | Methods: Study design and sampling, paragraph 2, 3 |
| Statistical methods | 12 | (*a*) Describe all statistical methods, including those used to control for confounding | Methods: data analysis |
|  |  | (*b*) Describe any methods used to examine subgroups and interactions | Methods: data analysis |
|  |  | (*c*) Explain how missing data were addressed | There were no missing data. |
|  |  | (*d*) If applicable, describe analytical methods taking account of sampling strategy | Not applicable |
|  |  | (*e*) Describe any sensitivity analyses | Not applicable |
| Results | | |  |
| Participants | 13* | (a) Report numbers of individuals at each stage of study—eg numbers potentially eligible, examined for eligibility, confirmed eligible, included in the study, completing follow-up, and analysed | Results: Prevalence of CL |
|  |  | (b) Give reasons for non-participation at each stage | Not applicable |
|  |  | (c) Consider use of a flow diagram | Not applicable |
| Descriptive data | 14* | (a) Give characteristics of study participants (eg demographic, clinical, social) and information on exposures and potential confounders | Results: Prevalence of CL, paragraph 1, 2 |
|  |  | (b) Indicate number of participants with missing data for each variable of interest | Not applicable |
| Outcome data | 15* | Report numbers of outcome events or summary measures | Results: Table 1, 2, 3; Figure 2 |
| Main results | 16 | (*a*) Give unadjusted estimates and, if applicable, confounder-adjusted estimates and their precision (eg, 95% confidence interval). Make clear which confounders were adjusted for and why they were included | Supporting information S2 Table  Results: Table 1, 2, 3  Results: Prevalence of CL, Environmental and host factors |
|  |  | (*b*) Report category boundaries when continuous variables were categorized | Not applicable |
|  |  | (*c*) If relevant, consider translating estimates of relative risk into absolute risk for a meaningful time period | Not applicable |
| Other analyses | 17 | Report other analyses done—eg analyses of subgroups and interactions, and sensitivity analyses | Results: Prevalence of CL, paragraph 2, 3  Results: Environmental and host factors |
| Discussion | | |  |
| Key results | 18 | Summarise key results with reference to study objectives | Discussion, paragraph 1, 2 |
| Limitations | 19 | Discuss limitations of the study, taking into account sources of potential bias or imprecision. Discuss both direction and magnitude of any potential bias | Discussion, paragraph 4 |
| Interpretation | 20 | Give a cautious overall interpretation of results considering objectives, limitations, multiplicity of analyses, results from similar studies, and other relevant evidence | Discussion, paragraph 1, 2, 3 |
| Generalisability | 21 | Discuss the generalisability (external validity) of the study results | Discussion, paragraph 4 |
| Other information | | |  |
| Funding | 22 | Give the source of funding and the role of the funders for the present study and, if applicable, for the original study on which the present article is based | Financial Disclosure section of submission. |

*Give information separately for exposed and unexposed groups.

**Note:** An Explanation and Elaboration article discusses each checklist item and gives methodological background and published examples of transparent reporting. The STROBE checklist is best used in conjunction with this article (freely available on the Web sites of PLoS Medicine at http://www.plosmedicine.org/, Annals of Internal Medicine at http://www.annals.org/, and Epidemiology at http://www.epidem.com/). Information on the STROBE Initiative is available at www.strobe-statement.org.
