# Supplementary material for: Prevalence and environmental determinants of cutaneous leishmaniasis in rural communities in Tigray, northern Ethiopia
Source: PLoS Negl Trop Dis. 2019 Sep 26;13(9):e0007722. doi: 10.1371/journal.pntd.0007722 (PMC6782111; doi:10.1371/journal.pntd.0007722)
Supplement: S1 Text — The form contains an introduction with basic details, a section for information on the household members, and a section with specific questions. (DOC) [file pntd.0007722.s002.doc]

# **S1 Text: Household Questionnaire for mapping cutaneous leishmaniasis in Tigray**

**Introduction**

**Instructions to enumerators**

- Greet the person you are interviewing and introduce yourself
- Identify your institution & explain the purpose of the study
- Ask if the respondent is willing to be interviewed. If they agree, start your interview; if not move to the next household.
- Interview the household head or a responsible person found in the house during the visit.
- Indicate that those with lesions have to sign a consent form
- Whenever observation is required, please do it carefully
- Write in the space provided, or circle the letter of the appropriate choice, or tick (**V**) as appropriate when you fill in the questionnaire.

**Household iden**tification

| **ID** | **Name** | **Code** |
| --- | --- | --- |
| Region |  |  |
| Zone |  |  |
| Woreda (district) |  |  |
| Tabia (subdistrict) |  |  |
| Kushet (village) |  |  |
| Name of Household Head |  |  |
| Occupation |  |  |
| House No |  |  |
| Name of respondent |  |  |
|  |  |  |

| Name of interviewer |  |
| --- | --- |
| Date of interview | **______/____ /______** |

**Section I. Household members & characteristics[[1]](#footnote-2)**

| **Number** | **1** | **2** | **3** | **4** | **5** | **6** |
| --- | --- | --- | --- | --- | --- | --- |
| Name  (Put HH head in No1) |  |  |  |  |  |  |
| Relation to HH head ***(1)***  (write code) |  |  |  |  |  |  |
| Sex  1 = M, 2= F |  |  |  |  |  |  |
| Age |  |  |  |  |  |  |
| Occupation |  |  |  |  |  |  |
| Highest grade of school completed  (write no or **00** if none) |  |  |  |  |  |  |
| Are skin lesions or scars present?  1=yes, 2=NO  ***(if NO, go to section II)*** |  |  |  |  |  |  |
| If yes, answer the following |  |  |  |  |  |  |
| Which?  1. scars, or  2. lesions, or  3. both |  |  |  |  |  |  |
| No (#) of scars or lesions present |  |  |  |  |  |  |
| Site of lesion / Scar **(2)**  1 = nose  2 =cheeks  3 = ears  4 = forehead  5 = limbs  6 = legs  7 = other |  |  |  |  |  |  |
| Duration of lesion |  |  |  |  |  |  |
| History of travel last 6 months before lesion was acquired  1= yes  2= NO |  |  |  |  |  |  |
| If yes, write place s/he traveled |  |  |  |  |  |  |

| ***(1) Codes***: | 01 = household head (HH) | 04 = grandchild | 07 = other relatives |
| --- | --- | --- | --- |
|  | 02 = wife or husband | 05 = parent | 08 = not related |
|  | 03 = son or daughter | 06 = brother or sister | 09 = don’t know |
| (**2)** If present at multiple sites, write all the respective numbers | | | |

**Section II. Specific questions**

**(**If there are more than one answer, circle all the possible answers)

| 2.1 | Type of living house | 1.stone walls; wood & earth roof  2.thatch  3. stone walls; corrugated iron sheet roof  4.1 & 2  5. 1&3  6.Other________________ |
| --- | --- | --- |
| 2.2 | Do the household members sleep outdoors? | 1. YES  2. NO  3. DON’T KNOW |
| 2.3 | Animal shed present? ***(Observe)*** | 1.YES  2. NO |
| 2.4 | If yes, is it separate from the main building?  ***(Observe)*** | 1.YES  2. NO |
| 2.5 | Do you or any member of your family living in the same compound own: | 1. Yes, 2. NO |
|  |  | Cattle: ________  Shots : ________  Pack: _________  Dogs: _________ |
| 2.6 | If yes to **Q2.5**, write their number | Cattle: ________  Shots : ________  Pack: _________  Dogs: _________ |
| **2.7.** | If dogs are present, do they have lesions on their ears? | 1. YES  2. NO  3. DON’T KNOW |
| 2.8 | Ever heard about CL? | 1. YES  2. NO  3. DON’T KNOW |
| 2.9 | If yes, is CL present in your village? | 1. YES  2. NO  3. DON’T KNOW |
| 2.10 | If yes, can you identify CL from other skin infections? | 1. YES  2. NO  3. DON’T KNOW |
| 2.11 | Which body part is most affected by CL? | 1. Face  2. Arms  3. Legs  4. Other ________________  5. Don’t know |
| 2.12 | Source of CL treatment? | 1. Traditional medicine  2. Modern medicine  3. DON’T KNOW |
| 2.13 | If traditional, what type of traditional treatment do you apply? | 1. Herbs  2. Hot iron or coal  3. Holy water or mud  4. Other_____________, specify  5. DON’T KNOW |
| 2.14 | If yes to Q2.8/2.9, do you know how CL is acquired or transmitted? | 1. ____________________  2. ____________________  3. ______________________  4. ____________________  5. ____________________  Don’t know |
| 2.15 | Is there any association between CL and hyraxes? | 1. YES  2. NO  3. DON’T KNOW |
| 2.16 | Are hyraxes present in your village? | 1. YES  2. NO  3. DON’T KNOW |
| 2.17 | If yes, distance in walking minutes? (ask the location & put your own estimate in meters) | __________minutes  ___________ meters |
| 2.18 | Do hyraxes eat crops? | 1. YES  2. NO  3. DON’T KNOW |
| 2.19 | Do you consider hyraxes as agricultural pests? | 1. YES  2. NO  3. DON’T KNOW |
| 2.20 | Are there caves nearby? **(Observe)** (ask the location & put your own estimate in meters) | 1. YES  2. NO  3. DON’T KNOW |
| 2.21 | If yes, how far is it from here (in walking minutes) (put your own estimate in meters) | __________minutes  ___________ meters |

1. This table has been reformatted for this publication. In the original form, up to 10 household members can be filled in and more space is available for providing answers. [↑](#footnote-ref-2)
